# Visual Nonclassical Receptive Field Effects Emerge from Sparse Coding in a Dynamical System

**DOI:** 10.1371/journal.pcbi.1003191

**Published:** 2013-08-29

**Authors:** Mengchen Zhu, Christopher J. Rozell

**Affiliations:** 1 Wallace H. Coulter Department of Biomedical Engineering, Georgia Institute of Technology, Atlanta, Georgia, United States of America; 2 School of Electrical and Computer Engineering, Georgia Institute of Technology, Atlanta, Georgia, United States of America; University of Tübingen and Max Planck Institute for Biologial Cybernetics, Germany

## Abstract

Extensive electrophysiology studies have shown that many V1 simple cells have nonlinear response properties to stimuli within their classical receptive field (CRF) and receive contextual influence from stimuli outside the CRF modulating the cell's response. Models seeking to explain these non-classical receptive field (nCRF) effects in terms of circuit mechanisms, input-output descriptions, or individual visual tasks provide limited insight into the functional significance of these response properties, because they do not connect the full range of nCRF effects to optimal sensory coding strategies. The (population) sparse coding hypothesis conjectures an optimal sensory coding approach where a neural population uses as few active units as possible to represent a stimulus. We demonstrate that a wide variety of nCRF effects are emergent properties of a single sparse coding model implemented in a neurally plausible network structure (requiring no parameter tuning to produce different effects). Specifically, we replicate a wide variety of nCRF electrophysiology experiments (e.g., end-stopping, surround suppression, contrast invariance of orientation tuning, cross-orientation suppression, etc.) on a dynamical system implementing sparse coding, showing that this model produces individual units that reproduce the canonical nCRF effects. Furthermore, when the population diversity of an nCRF effect has also been reported in the literature, we show that this model produces many of the same population characteristics. These results show that the sparse coding hypothesis, when coupled with a biophysically plausible implementation, can provide a unified high-level functional interpretation to many response properties that have generally been viewed through distinct mechanistic or phenomenological models.

## Author Summary

Simple cells in the primary visual cortex (V1) demonstrate many response properties that are either nonlinear or involve response modulations (i.e., stimuli that do not cause a response in isolation alter the cell's response to other stimuli). These non-classical receptive field (nCRF) effects are generally modeled individually and their collective role in biological vision is not well understood. Previous work has shown that classical receptive field (CRF) properties of V1 cells (i.e., the spatial structure of the visual field responsive to stimuli) could be explained by the sparse coding hypothesis, which is an optimal coding model that conjectures a neural population should use the fewest number of cells simultaneously to represent each stimulus. In this paper, we have performed extensive simulated physiology experiments to show that many nCRF response properties are simply emergent effects of a dynamical system implementing this same sparse coding model. These results suggest that rather than representing disparate information processing operations themselves, these nCRF effects could be consequences of an optimal sensory coding strategy that attempts to represent each stimulus most efficiently. This interpretation provides a potentially unifying high-level functional interpretation to many response properties that have generally been viewed through distinct models.

## Introduction

As we seek to understand how sensory nervous systems process information about their environment, one of the most common quantitative descriptors of neural coding has been the notion of a classical receptive field (CRF) [1]. In general, the CRF is a measurement of the portion of the stimulus space that causes a change in a neuron's response when a stimulus is presented (or removed). For example, beginning with the pioneering work of Hubel and Wiesel [2], simple cells in the primary visual cortex (V1) have been characterized as feature detectors with CRFs that are selective for location, orientation and spatial frequency.

Unfortunately, a simple linear-nonlinear model based on the measured CRF (e.g., linear filtering with the CRF followed by nonlinear thresholding or saturation) is insufficient to explain many response properties of V1 cells. For example, extensive electrophysiology studies have shown that many V1 simple cells also receive contextual influence where stimuli not part of the CRF can modulate the cell's response to CRF stimuli (reviewed in [3]). Furthermore, when driven by rich stimuli within the CRF, simple cells exhibit complex nonlinear response properties that cannot be captured by thresholding or saturation alone [4]. We use the term *non-classical receptive field (nCRF) effects* to collectively refer to these contextual modulations and nonlinear response properties.

Understanding nCRF effects is likely critical for understanding the coding of natural stimuli because they arise under stimulus conditions that are more complex and ecologically relevant than the stimuli often used in CRF mapping experiments (e.g., sinusoidal gratings, white noise, sparse dots). Indeed, recent electrophysiology experiments with natural video stimuli have shown contextual influence in V1 responses [5]–[8]. Furthermore, observed V1 nCRF effects have been related to perceptual contextual effects such as contour integration [9].

Given the wide range of different nCRF effects reported in the literature, it is still unclear how these effects are related or what collective role they play in sensory coding. Many individual nCRF effects have been successfully described in terms of potential underlying circuit mechanisms (i.e., mechanistic models, reviewed in [10]) or compact stimulus/response descriptions (i.e., phenomenological models, reviewed in [3]). While valuable, these approaches do not fully address the functional significance of nCRF effects or illuminate their role in sensory information processing. In another direction, individual nCRF effects have also been connected to potential benefits in specific tasks (e.g., curvature detection [11], contour integration as reviewed in [12], figure-ground segregation as reviewed in [13]). While these approaches are also valuable, these types of models have limited explanatory power because they only address narrow subsets of biological vision (i.e., individual tasks) and they do not show that the processing strategies represented by nCRF effects are optimal for the given tasks. In short, models constructed for individual effects do not connect this broad range of response properties to the optimal sensory coding strategies that can provide a parsimonious description in terms of fundamental system goals.

One central goal of theoretical and computational biology is to provide functional insight into biological phenomenon by using high-level models (often abstracting away specific experimental detail) to generalize and explain disparate observations. Regarding CRF properties in biological vision, one model that has had success in this regard is the sparse coding hypothesis. Sparse coding conjectures an optimal coding goal where a population of cells encodes a stimulus at a given time using as few active units as possible. Specifically, the model of interest optimizes *population* sparsity, which is distinct from *lifetime* sparsity (a single cell being active a small fraction of the time). In seminal results, the high-level sparse coding model (combined with unsupervised learning using the statistics of natural images) has been shown to be sufficient to explain the emergence of V1 CRF shapes both qualitatively [14] and quantitatively [15]. In addition to this success providing functional insight into CRF properties, distributed sparse neural codes have many potential benefits (e.g., explicit information representation and easy decodability at higher processing stages [16], metabolic efficiency [17], increased capacity of associative and sequence memory models [18], [19]) and are consistent with many recent electrophysiology experiments [20].

Despite the success accounting for the emergence of CRF properties, there has been little work showing that sparse coding can account for response properties observed in V1 cells. There have been several recent experimental results showing that stimuli in the CRF surround can cause individual cell responses with higher lifetime sparsity than expected (e.g. [5], [6], [8], reviewed in [21]). While this experimental observation provides encouraging support for the sparse coding hypothesis, it does not imply that a sensory coding model optimizing sparsity is sufficient to account for V1 response properties (including nCRF effects). Sparse coding is one interpretation of the efficient coding hypothesis [22] (conjecturing that neural coding should successively remove stimulus redundancy), and other models related to efficient coding have shown individual model cells that produce some nCRF effects (reviewed in detail in the Discussion section). However, few of these models have shown the broad spectrum of observed nCRF effects in single cells, and none have yet demonstrated the diversity of population response properties reported in the literature for a single effect. Taken together, the evidence of sparsity in experimental observations and the prior success of other related models gives motivation for investigating the potential role of sparse coding in producing nonclassical response properties.

In this paper we demonstrate that a wide variety of nCRF effects are emergent properties of a sparse coding model implemented in a neurally plausible network structure. Specifically, we use the experimental paradigms described in the literature for a wide variety of nCRF effects (e.g., end-stopping, surround suppression, contrast invariance of orientation tuning, cross-orientation suppression, etc.) to replicate these electrophysiology experiments on a dynamical system implementing optimal sparse coding. In the first contribution of this paper, we show that this model produces individual units that reproduce a wide variety of canonical nCRF effects. While another recent model [23] has also shown nearly all of these effects in a unified model along with some increased sparsity of the responses, the present work is the first to show that these effects can arise in a model that has only sparsity as the coding objective. In the second contribution of this paper, when the population diversity of an nCRF effect has been reported in the literature (either through population statistics or multiple individual cells with varying response properties), we also show that this simulated population demonstrates much of the same population heterogeneity reported in the literature. Notably, the results we report are produced with a single set of model parameters (i.e., parameters are not tuned to produce each different effect), despite the system only being designed to optimize sparsity and not constructed to produce nCRF effects. These results show that the sparse coding hypothesis, when coupled with a biophysically plausible implementation, can provide a unified high-level functional interpretation to many population response properties that have generally been viewed through distinct models.

## Results

### Sparse coding and dynamical systems

The sparse coding model proposes that V1 encodes an image patch 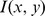 with 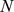 pixels as approximately a linear superposition of 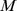 (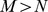) dictionary elements 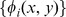,
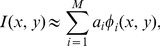
(1)where the coefficients 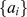 represent the population activity (e.g., average firing rates) [14]. In this model, a neural population encoding the image 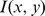 would calculate activity levels 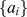 that minimize an energy function that is a weighted combination of a data fidelity term (e.g., mean-squared error) and a sparsity penalty (e.g., the coefficient magnitudes),
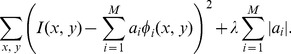
(2)Here 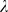 is a system parameter that controls the trade-off between the fidelity of the representation and the sparsity of the coefficients.

The sparse coding model is a functional model that can be implemented through many different mechanisms, including using generic convex optimization algorithms designed for digital computers. In this study we use a dynamical system proposed in [24] that employs neurally plausible computational primitives. Specifically, we implemented the sparse coding model by simulating the dynamical system given by:
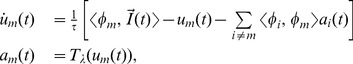
(3)where 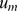 is an internal state variable for each node (e.g., membrane potential), 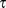 is the system time constant, and 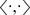 is an inner product over the spatial dimensions. In the system dynamics, 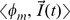 captures the feedforward filtering while 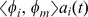 captures the recurrent interactions that implement competition between cells to represent the stimulus. Note that the recurrent interaction between those cells is inhibitory if 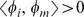 and excitatory if 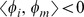 (since 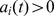 in our model). 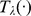 is the soft thresholding function:
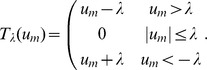
The input stimulus can be changed dynamically (e.g., a drifting sinusoidal grating), in which case the time-varying coefficients 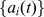 will track approximate solutions, with the solution accuracy determined by the time scale of the input changes relative to the system dynamics. We note that recent theoretical work has demonstrated several network architectures that can efficiently implement other versions of sparse coding with various degrees of biological plausibility [15], [25], [26]. The network architecture being used in this study provably solves the optimization in Eq. (2) with strong convergence guarantees [27], can implement many variations of the sparse coding hypothesis (i.e., different sparsity-inducing cost functions) [28], and is implementable in neuromorphic analog circuits [29].

**Figure 1:**
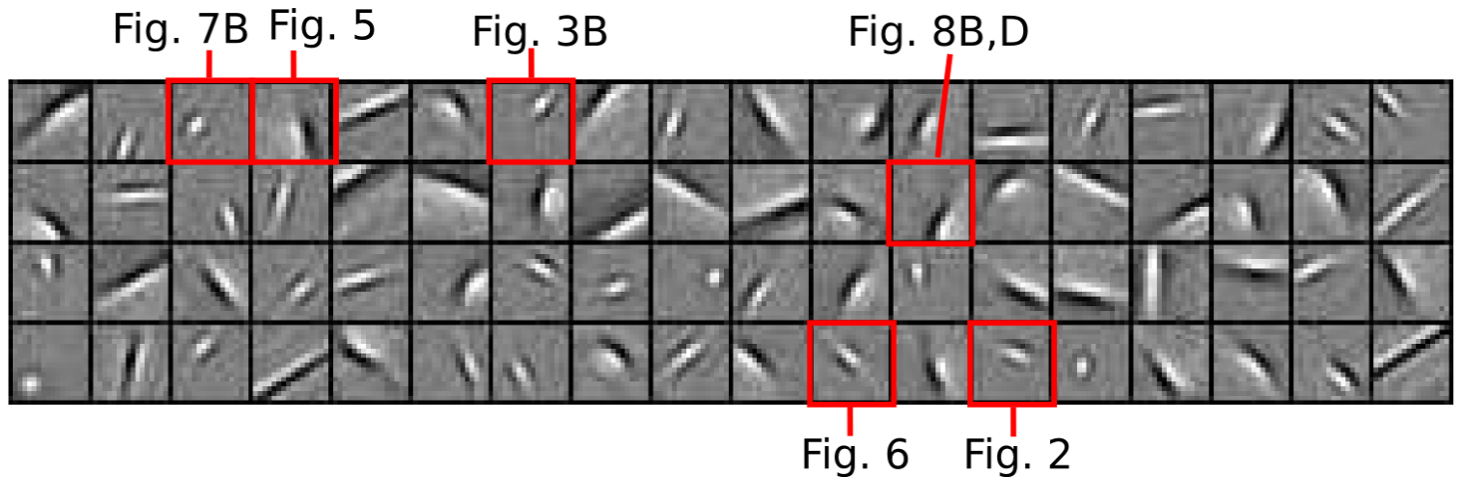
Subpopulation of dictionary elements (“CRFs”) studied. The 72 dictionary elements that were recorded from in the model simulation. Dictionary elements were optimized for sparse coding under natural scenes (as described in the text) and selected for well-localized CRFs in the image patch. The units whose single cell activities are presented in later figures are indicated by red rectangles.

In our implementation, a dictionary 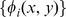 optimized for sparse coding with natural scenes was determined via unsupervised learning under sparsity constraints using whitened natural scenes as the training set (whitening is a first-order approximation of retinal processing). The learned dictionary was overcomplete with 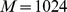 effective dictionary elements for the 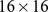 pixel image patches used as stimuli. The training set, whitening and learning rule were all exactly as in [14], while the sparse codes during training (i.e., solutions to (2)) were calculated using a standard software package [30] (for computational efficiency) with 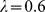. We interpret these dictionary elements as the classical spatial receptive fields (CRFs) of the simulated neurons. This interpretation is supported by our own simulated receptive field mapping experiment (results not shown) using sparse dot stimuli, similar to previous studies (e.g., see Fig. 4b in [14]). The results demonstrated in this study are based on the responses of 72 units in this dictionary that had CRFs well-localized within the available image patch (shown in Fig. 1).

The system parameters described above (i.e., membrane time constant, sparsity level 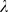) are kept the same for every simulation in this paper (details given in Materials and Methods). In other words, no attempt was made to tailor the system to reproduce each effect individually (some interesting exceptions where parameter changes correspond to apparently conflicting results in the literature are shown in the Supporting Information). We interpret the sparse coefficients 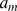 in Eq. (2) as the trial-averaged instantaneous spike rate of neurons in the model population. To do this, we also impose a positivity constraint 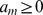 and extend the dictionary matrix by including both the original dictionary elements and the negative of the dictionary elements (i.e., doubling the size of the matrix to use the same effective dictionary as if there were both positive and negative coefficients). This mirrored receptive field structure is reminiscent of the push-pull feedforward input structure in the visual simple cells [31].

In the following sections, we highlight several common nCRF effects from the literature and illustrate that this sparse coding model can largely reproduce both reported individual response properties and much of the reported response diversity across V1 neurons. For each nCRF effect the simulation was constructed to match as closely as possible the experimental protocol described in the experimental procedures section of the corresponding electrophysiology paper, including stimulus construction parameters and data analysis (details given in Materials and Methods). We classify the studied nCRF effects into three groups: suppressive effects that are evoked by the presence of stimuli outside the classical receptive field (CRF surround effects), effects where the response modulation depends on the orientation of the stimulus in the surround (CRF surround orientation effects) and effects that reflect the nonlinearity of the CRF center (nonlinear CRF effects).

### CRF surround effects

**Figure 2:**
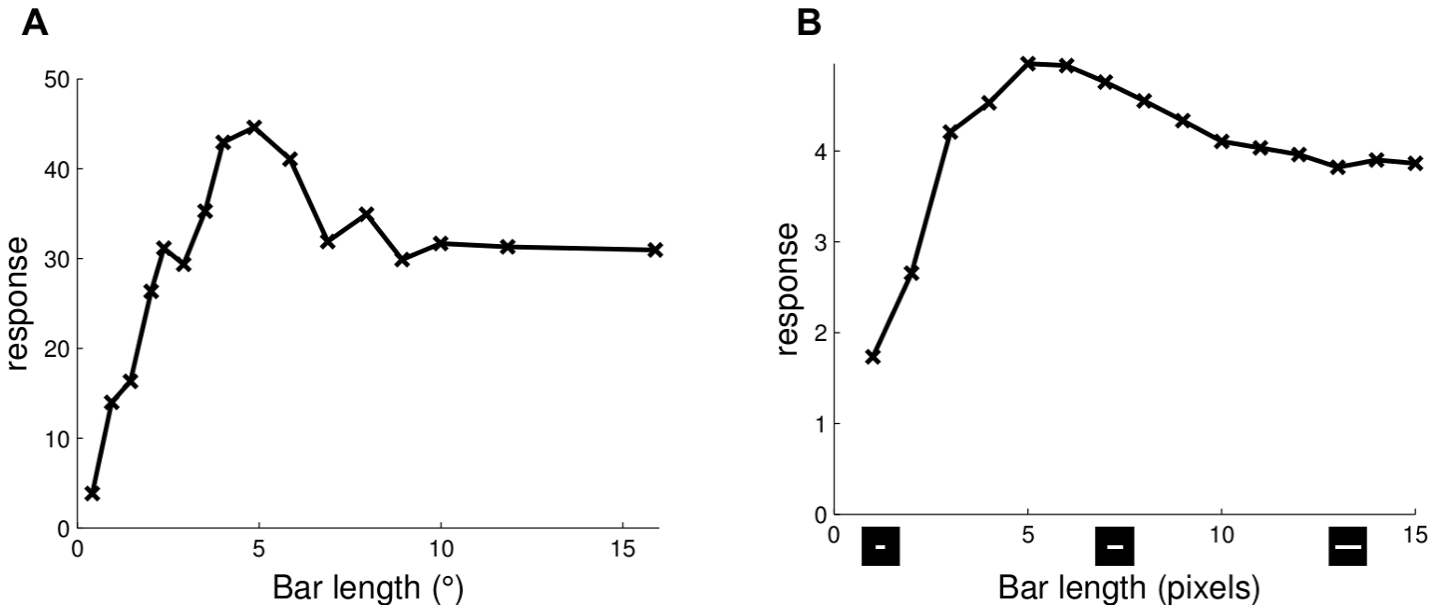
End-stopping. (A) End-stopping response in a simple cell from cat V1 responding to an optimally-oriented light bar stimulus (data replotted from [32], Figure 1). (B) The length tuning curve of a simulated sparse coding model neuron (target) demonstrates end-stopping behavior.

Stimuli in the region surrounding the CRF can have a modulatory effect on a neuron's response despite not inducing significant response in isolation (by definition of the CRF). In perhaps the simplest form of this suppressive modulation, it has long been known that some V1 neurons exhibit *end-stopping* where the spike rate decreases for a cell responding to an optimally-oriented bar stimulus when the bar length is increased beyond the CRF boundaries. An example figure depicting the end-stopping effect as observed in cat electrophysiology recordings [32] is reproduced in Fig. 2A. When simulating this experiment [32] on the sparse coding model, some of the model cells (such as the target cell shown in Fig. 2B) exhibit the same characteristic suppression with increasing bar length. The end-stopping effect was previously shown in [33] to emerge in the sparse coding model. The end-stopping effect can be simply understood in terms of the goals of sparse coding. When the bar is short, the CRF of the target cell is the most efficient description of the stimulus and that cell has the strongest response. However, when the bar is long enough that it is better explained by the CRFs of other cells, the target cell becomes suppressed by these competitors so as to maintain a sparse representation. The Discussion section contains a detailed look at how the network interactions supporting the sparse coding model can produce this effect.

**Figure 3:**
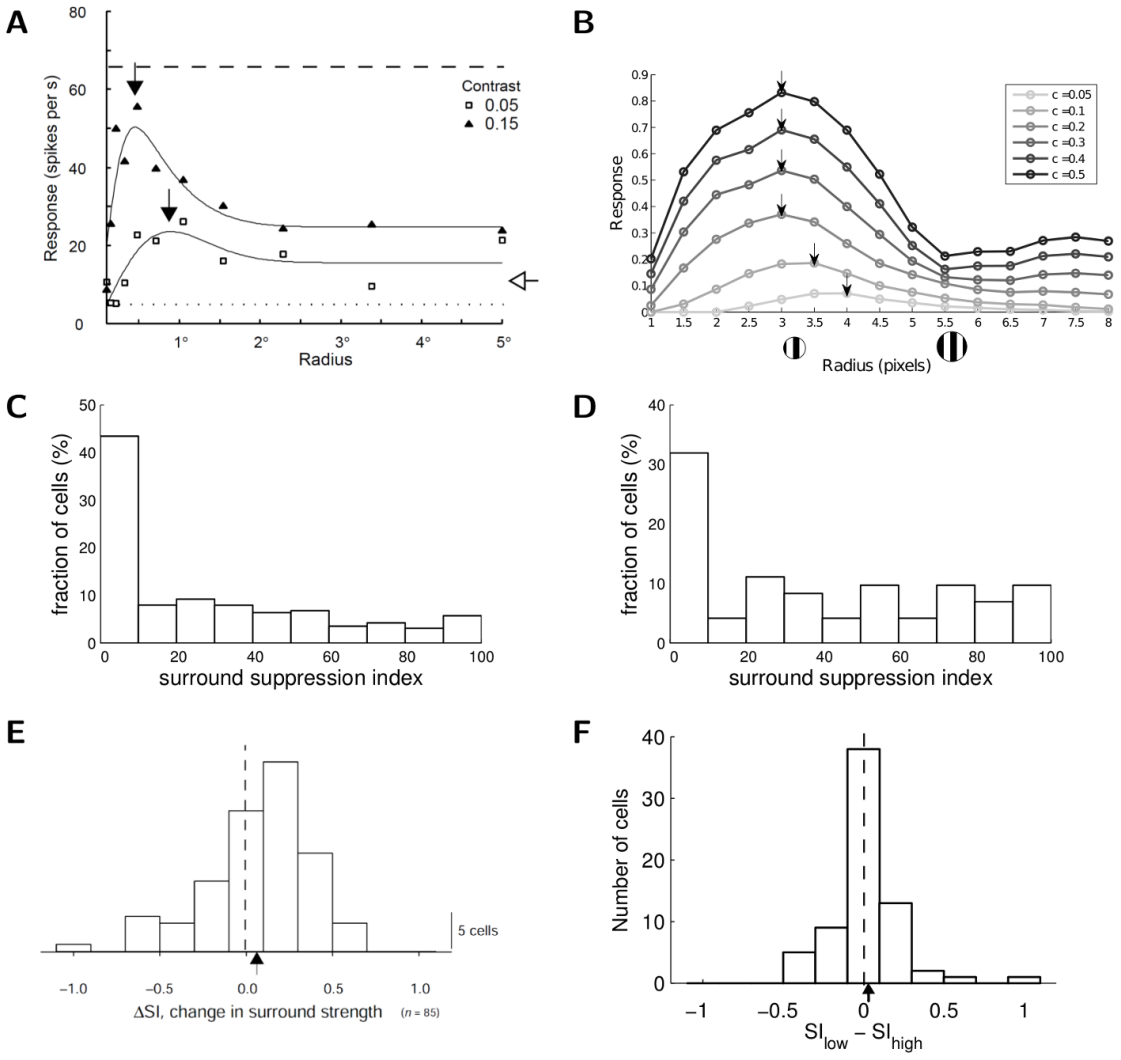
Surround suppression and RF expansion. (A) A plot illustrating that cortical neurons show surround suppression and expansion of CRF size at low contrast (reprinted by permission from Macmillan Publishers Ltd: Nature Neuroscience, Figure 1a from [37]). (B) The size tuning curve of a simulated sparse coding model neuron at various contrast levels (“c” stands for contrast, with lighter curves representing lower contrast). The model neuron exhibits two characteristic behaviors reported in the electrophysiology literature: suppression with increasing stimulus size and an increase in the optimal stimulus size with lower contrast. The maximum of each tuning curve is marked by an arrow. (C) Physiologically measured distribution of surround suppression index (SI) in cat V1 (data replotted from [36], Figure 2A), illustrating that most cells do not exhibit significant surround suppression and the SI distribution is relatively uniform among suppressive cells. (D) The SI distribution for the model cells, illustrating the same qualitative properties as the distribution in (C). (E) Distribution of the SI difference (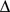
SI) between low and high contrast levels in macaque V1 (reprinted by permission from Macmillan Publishers Ltd: Nature Neuroscience, Figure 6b from [37]). The mean difference is 0.06, demonstrating that on average the SI for a cell is contrast invariant. (F) The distribution of 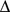
SI for the sparse coding model cells. The mean difference is 0.02, also demonstrating contrast invariance in SI.

Similar to end-stopping, some V1 neurons also exhibit *surround suppression* where their response to a sinusoidal grating patch decreases as the patch size increases beyond the CRF. Additionally, the tuning curve for patch size often exhibits *receptive field expansion* at low contrast, meaning that the patch size achieving the maximum response increases at low contrast (Fig. 3A). As illustrated in the response of an example model cell shown in Fig. 3B, the sparse coding model can exhibit the same basic suppression and receptive field expansion properties observed in electrophysiology experiments. In addition, we note that the slight increase of response level (i.e., response rebound) at large stimulus size visible in Fig. 3B is also visible in Fig. 3A and discussed elsewhere [34].

The network interactions giving rise to surround suppression are presumably similar to that of end-stopping, but are more difficult to specify given the added dynamics of the drifting grating stimulus. In particular, due to the suboptimal match of the target CRF to the larger stimulus, competition from other cells (that better match the larger stimulus) can suppress the target cell's response. This competition can also be modulated by the stimulus contrast and may contribute to the receptive field expansion. Specifically, at low contrast the competing cells have lower response levels (resulting in a weaker suppressive effect on the target cell), enabling the response of the target cell to grow with the stimulus size.

Despite the evidence detailed above that some biological and model V1 neurons exhibit surround suppression, a single example cell is insufficient to quantify the prevalence of this effect in a population encoding sensory information. While many nCRF effects are reported as single cell response properties, some studies have attempted to quantify how strongly an effect is expressed across the population. In the case of surround suppression, two metrics have been used to quantify the degree of suppression and receptive field expansion demonstrated by a cell. One is the suppression index (SI), calculated as the ratio between the (suppressed) response value at large stimulus sizes and the peak response value (indicated by arrows in Fig. 3B). The second metric is the RF expansion ratio, calculated as the ratio of the size tuning peak location at high contrast against that at low contrast.

In many physiological studies (both in monkeys [35] and in cats [36]), a large proportion of cells actually show little suppression, with relatively few cells exhibiting strong suppression. An example SI distribution from cat V1 is shown in Fig. 3C, demonstrating a dominant peak at zero suppression and a relatively uniform distribution among more suppressive cells. A similar population distribution emerges from the sparse coding model cells, as illustrated in Fig. 3D. Another characteristic of the surround suppression index is that it is largely invariant to the stimulus contrast. In other words, the difference in SI at high and low contrast is close to zero (Fig. 3E) with a mean value of 0.06 [37]. We also observed this characteristic in the sparse coding model cells (Fig. 3F), with a mean SI difference of 0.02. We note here that some studies (e.g. [38]) recorded unusually high percentage of cells showing significant surround suppression, perhaps due to a different experimental preparation. Interestingly, the sparse coding model can qualitatively reproduce these apparently conflicting results by using a different set of parameters to encourage more sparsity (see Fig. S2 which is described in Supporting Information Text S1).

**Figure 4:**
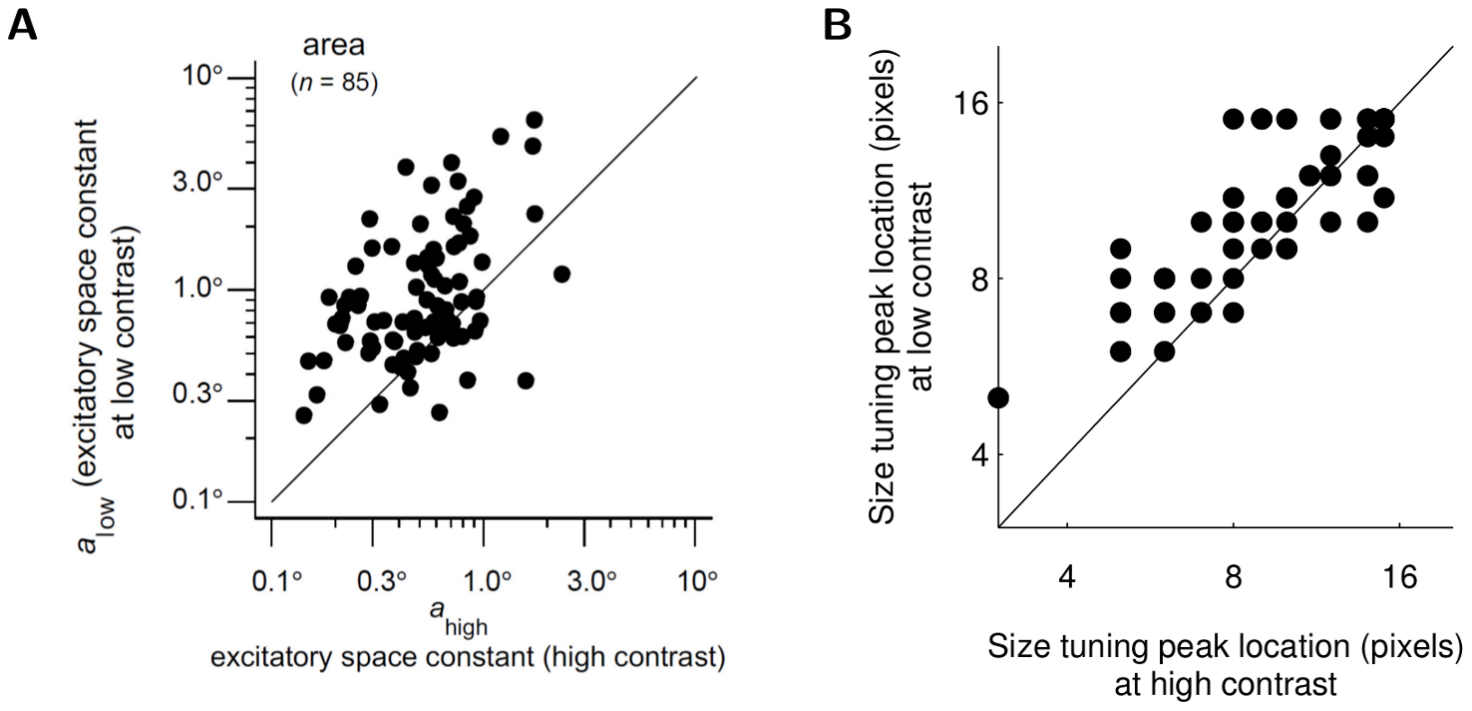
Size tuning peak at high vs. low contrast. (A) RF expansion of macaque V1 cells (reprinted by permission from Macmillan Publishers Ltd: Nature Neuroscience, Figure 3a from [37]). (B) RF expansion of sparse coding model cells. Most points lie above the diagonal, indicating that (on average) the optimal stimulus size is larger at lower contrasts and the cell demonstrates RF expansion.

A scatterplot of RF expansion ratios for V1 cells in macaque [37] shows clearly that on average, the CRF size is larger at low contrast than at high contrast (Fig. 4A). A scatterplot of expansion ratios for the sparse coding model population shows the same qualitative trend of expanding CRF size at low contrast. We note that the mean expansion ratio in the sparse coding model cells (1.16) is lower than typically reported values in the electrophysiology literature (e.g., 2.3 in [37]). This quantitative difference may be due to variations in the RF expansion ratio definitions (e.g., the study in [37] uses a difference of Gaussians fit rather than tuning curve peaks), the lack of contrast saturation in the present model (see Discussions), or biased sampling of neurons in the electrophysiology literature [39]. The possibility that the true expansion ratio might be lower than previously reported is corroborated by a recent study reporting that as many as 40% of cat V1 neurons show length tuning peaks that are invariant to contrast changes [40].

### CRF surround orientation effects

**Figure 5:**
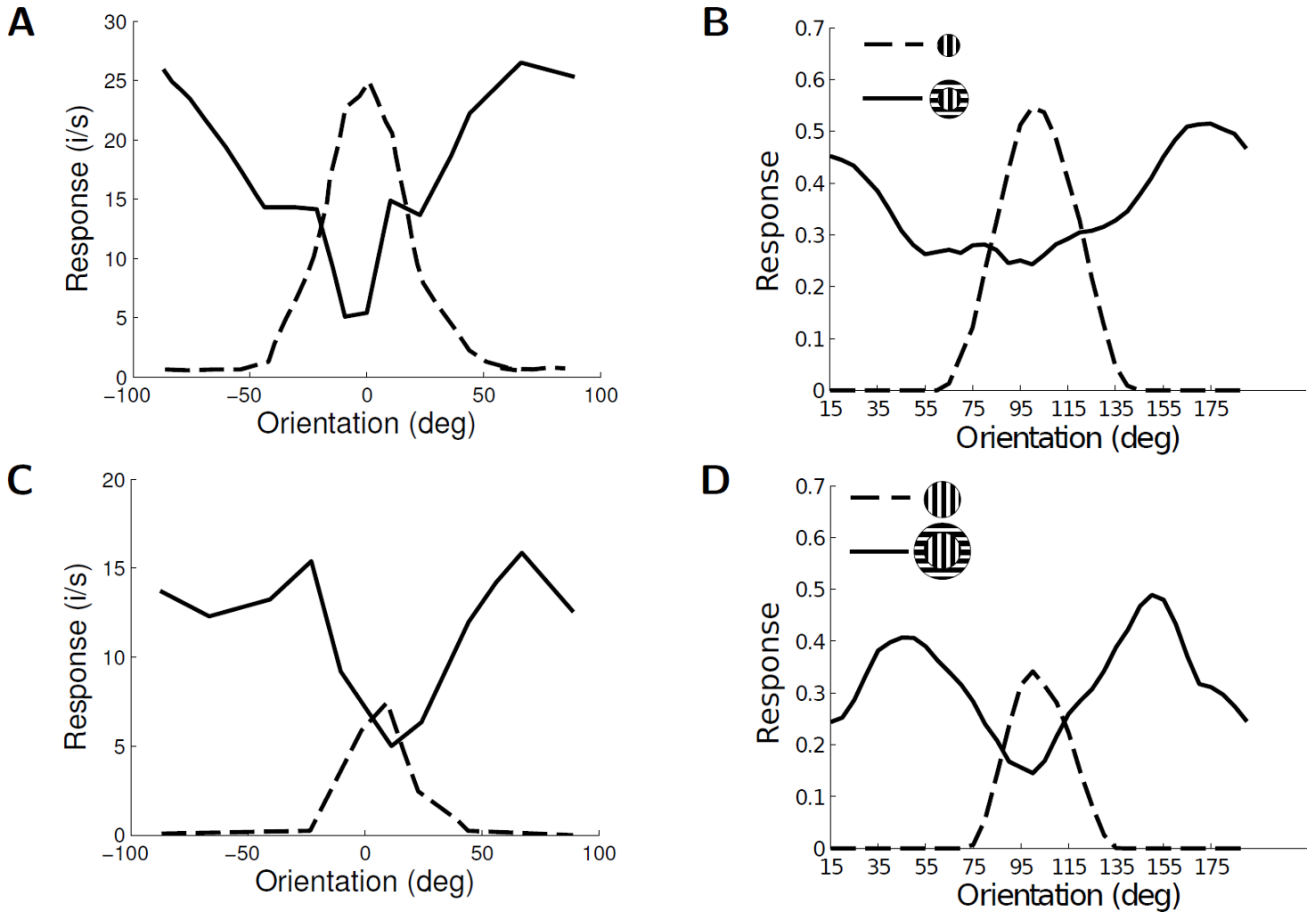
Orientation tunings for surround suppression and facilitation. (A) Center and surround tunings with the optimal stimulus center size in macaque V1 (data replotted from [41], Figure 2A). The center orientation tuning curve (dashed line) shows the cell's response to a CRF sinusoidal grating. With the CRF stimulus fixed to an optimally-oriented grating, the surround orientation tuning curve (solid line) shows the cell's response to a sinusoidal grating in the annular surround at various orientations. (B) A sparse coding model cell demonstrating similar surround orientation tuning properties, with highest levels of suppression at iso-oriented surround stimuli and almost no suppression for ortho-oriented surround stimuli. (C) Center and surround orientation tunings of the same cell as in (A) with the stimulus center size increased beyond the CRF and the width of the surround annulus unchanged (data replotted from [41], Figure 2B). (D) The same sparse coding model cell as in (B) demonstrates the facilitatory effects at ortho-oriented stimuli seen in (C).

The modulatory effects seen from surround stimulation can depend on a number of stimulus properties, including contrast, spatial extent (relative to the CRF), and stimulus orientation in the surround. In particular, modulation is often most suppressive when the surrounding stimuli are at orientations parallel to the preferred CRF orientation (iso-oriented), and less suppressive (or even facilitatory) when the stimuli are perpendicular to the preferred CRF orientation (ortho-oriented). For example, when stimulating a cell with an optimally oriented sinusoidal grating just covering the CRF (i.e., the orientation eliciting the strongest response), a grating in the annulus surrounding the CRF often suppresses the cell when it is iso-oriented and has little effect when it is ortho-oriented. An example of this *surround orientation tuning* in macaque V1 cells [41] is shown in Fig. 5A. The sparse coding model cells can also demonstrate the same type of surround orientation tuning, as illustrated by the model cell response shown in Fig. 5B. This tuning behavior in the model is likely due to the difference in the strength of competition with different stimulus surround orientations. In particular, the competing cells stimulated by iso-oriented surrounds are likely to have stronger CRF overlaps with the target cell and therefore induce more competition than the cells stimulated by ortho-oriented surround stimuli.

Orientation tuned surround effects can have substantial variations, even with minor changes in the stimulus. For example, the modulatory effect can be facilitatory at some surround orientations, causing a net increase in the response of the cell to CRF stimulation alone. This facilitatory effect is often seen when using a center stimulus slightly larger than the optimal size [41], as shown in Fig. 5C for the same cell as in Fig. 5A. Interestingly, increasing the size of the center stimulus for a model cell can likewise induce facilitation when the surround stimulus is close to ortho-oriented (shown in Fig. 5D for the same cell as in Fig. 5B).

As with surround suppression, a single example of facilitation in the surround orientation tuning does not characterize the prevalence of this effect in a population of V1 cells encoding a stimulus. The degree of facilitation expressed by a neuron can be characterized by measuring the ratio between the maximum of the surround orientation tuning (the maximum of the solid line in Fig. 5B) and the response to the center at the optimal orientation with no surrounding stimulus (the maximum of the dashed line in Fig. 5B). In macaque V1 [42], the median of the facilitation ratio across the measured population was found to be 1.44 at high contrast and 1.71 at low contrast. The sparse coding model cells show a similar dependency on contrast levels, with the median facilitation ratio ranging from 1.15 at high contrast and 1.31 at low contrast.

**Figure 6:**
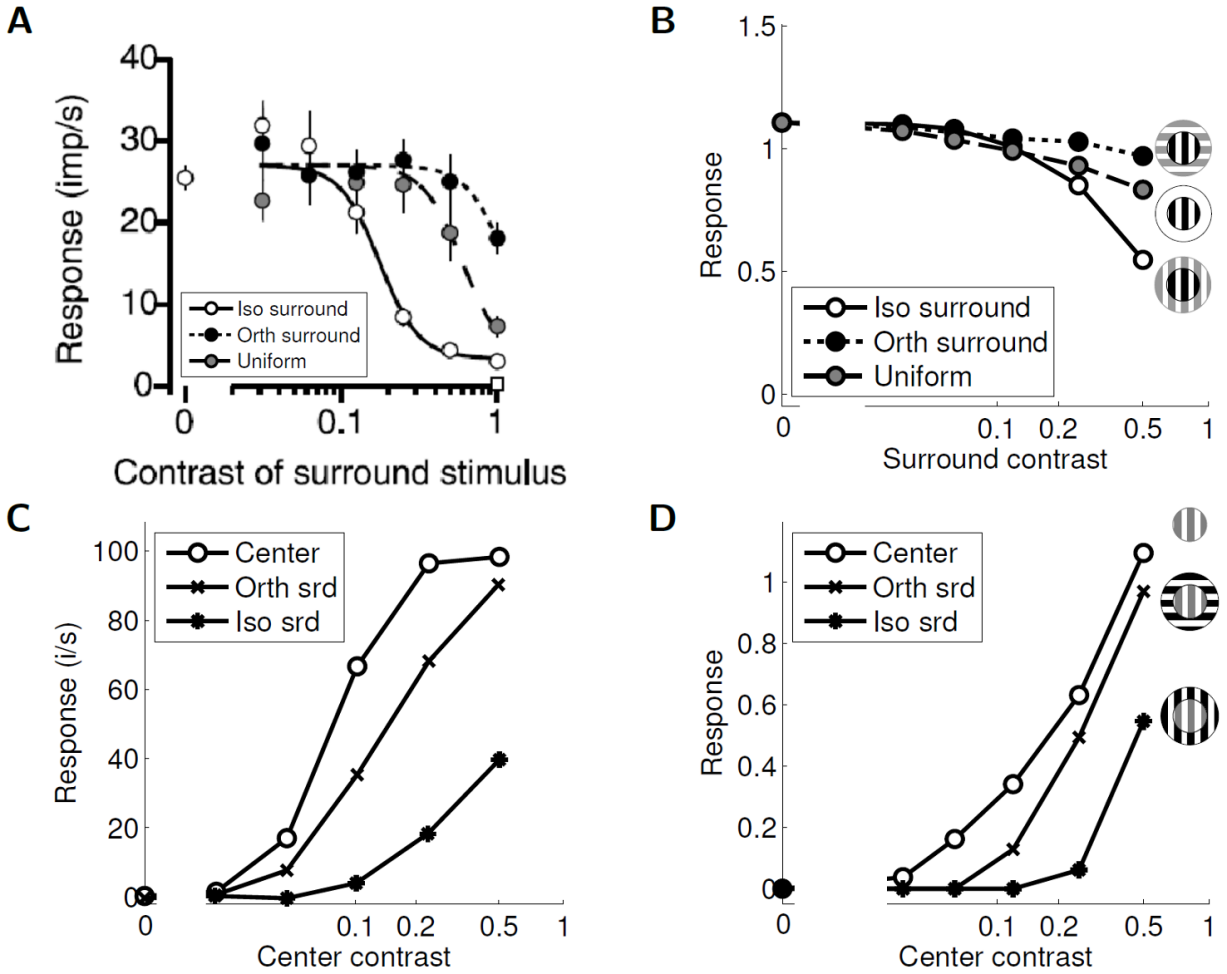
The effect of contrast on surround influences. (A) Surround contrast tunings with fixed center contrast in macaque V1 and varying surround stimuli (reprinted by permission from the Society for Neuroscience: The Journal of Neuroscience, Figure 6B from [35]). The gray markers correspond to responses to a uniform surround at different contrast. (B) Surround contrast tunings with fixed center contrast in the sparse coding model. As with the neuron responses in (A), the model cell is most suppressed for iso-oriented surround stimuli at high contrast. (C) Center contrast tunings with fixed surround contrast in macaque V1 simple cells with varying surround orientations (data replotted from [43], Figure 5A). (D) Center contrast tunings with fixed surround contrast in the sparse coding model. As with the neuron responses in (C), the model cell shows that (especially at high contrast) an iso-oriented surround (asterisk markers) is more effective than an orthogonal surround (cross markers) at suppressing the response to the center alone (white circle markers). As mentioned in the text (see Discussions), the lack of contrast saturation in the present sparse coding model is evident in this figure by the model response at high contrast.

The surround orientation tuning properties described above can be substantially influenced by the contrast difference between the center and the surround. For example, if the center contrast is fixed and the surround contrast is varied, the most significant suppression in individual macaque neurons was observed with the iso-orientated stimuli at high surround contrast (see Fig. 6A) [35]. Similarly, when plotting the responses as a function of center contrast for various surround settings (e.g., no surround, iso-oriented, and ortho-oriented), the suppressive effects in macaque were most pronounced with the iso-oriented stimuli at high center contrast (see Fig. 6C) [43]. Both of these dependencies on contrast can also be observed in the sparse coding model cells, as shown in Fig. 6D and Fig. 6B. Again we note that in some physiological studies an apparently conflicting result is reported where cat V1 neurons show facilitation with iso-oriented surround stimuli at low CRF contrast [44] (Fig. S3A which is described in Supporting Information Text S1). Interestingly, the sparse coding model can also reproduce this behavior when using a different set of parameters (see Fig. S3B which is described in Supporting Information Text S1).

### Nonlinear CRF effects

**Figure 7:**
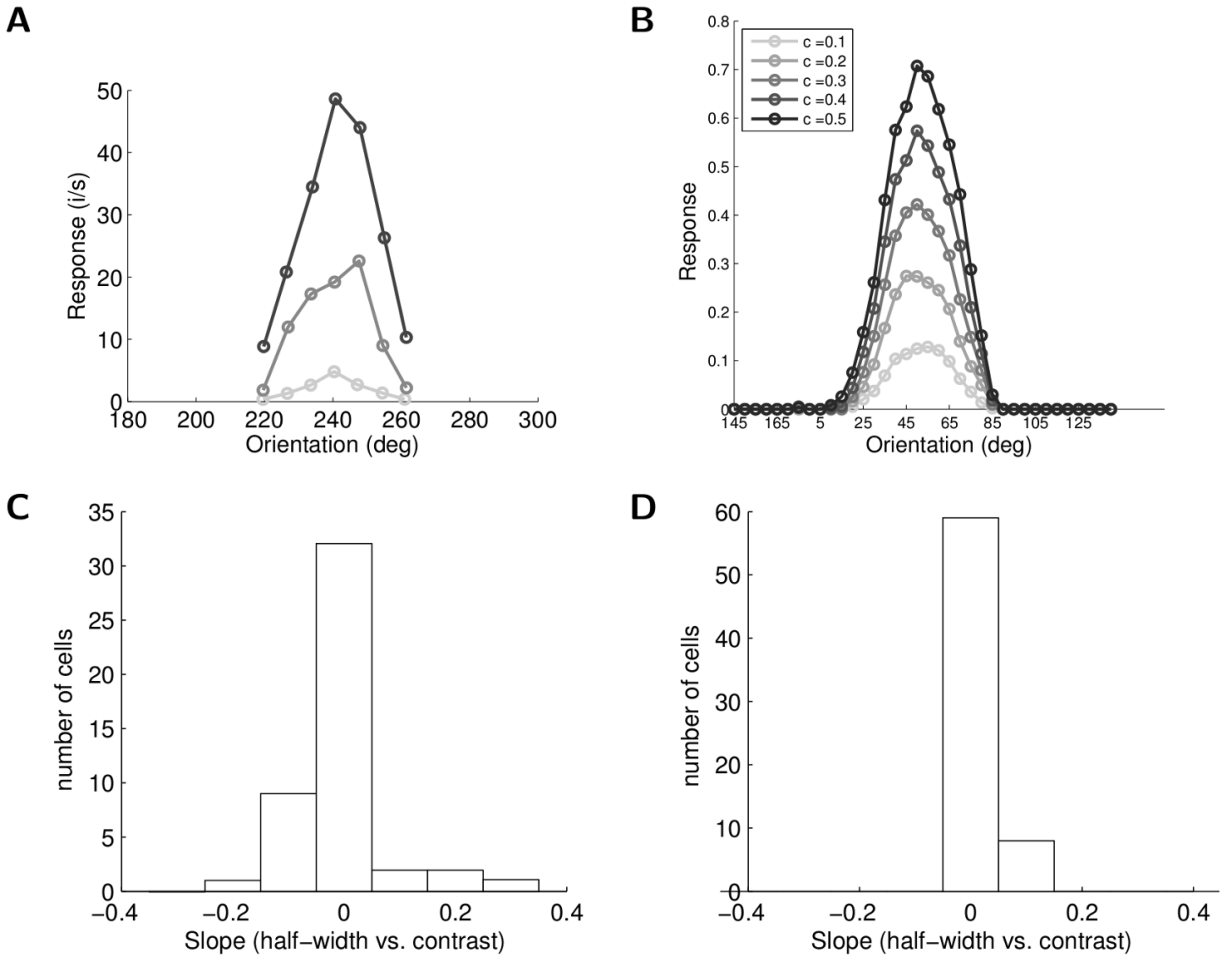
Contrast invariant orientation tuning. (A) Contrast invariance of orientation tuning curves recorded in cat V1 (data replotted from [46], Figure 3A). Note that the width of the orientation tuning curve does not change with contrast. (B) Sparse coding model neuron that demonstrates the same invariance property. Lighter curves correspond to lower contrast (“c” denotes contrast level). (C) Distribution of the slope of tuning curve half-width vs. the contrast in ferret V1 (data replotted from [47], Figure 3B). The sharp distribution around 0 indicates that the tuning curve half-width is contrast invariant (mean value is 0.002). (D) Distribution of the half-width vs. the contrast slope in the sparse coding model cells (mean value is 0.032). The model cells clearly demonstrate contrast invariance of the tuning curve half-width, and an even tighter peak around zero slope than shown in (C).

Even when the stimulation is confined to the CRF with no involvement of the surround, cells in V1 exhibit several nonlinear effects that cannot be explained by a canonical linear-nonlinear model [4]. One example of such an effect is the *contrast invariance of orientation tuning* for V1 cells. In a linear-nonlinear model based on CRFs, higher contrast stimuli evoke stronger responses that more readily exceed the spiking threshold, thus broadening the orientation tuning curve for higher contrast stimuli (the “iceberg effect” [45]). However, as reported in the cat physiology literature, the orientation tuning width is largely contrast invariant [46] as demonstrated in Fig. 7A. Cells from the sparse coding model can also display this contrast invariance in the width of their orientation tuning curves, as shown in Fig. 7B. This invariance can potentially be attributed to recurrent inhibition from competing cells at orientations where the target cell is not the most efficient description (e.g., ortho-oriented stimuli). Even though these competing cells may not have large overlap with the CRF of the target cell, as the contrast increases they will become more active and induce stronger inhibition, thereby narrowing the tuning width of the target cell compared to the low-contrast response. Indeed, compared to the predictions of a linear-nonlinear model (not shown), the tuning width from our model is much narrower.

The degree to which the width of the orientation tuning curve changes for a cell can be quantitatively measured by calculating the half-width at half-height of the Gaussian fit to the tuning curve for various contrast levels [47]. The population statistics can be plotted as a histogram tabulating the slope of the best linear fit to the width expansion with contrast for each cell. An example of this measure from ferret V1 demonstrating that the tuning curve width is almost constant with contrast is shown in Fig. 7C [47]. In this same measure, the sparse coding model also exhibits strong contrast invariance properties across the population, as shown in Fig. 7D. Both the ferret V1 population and the sparse coding model have a slope tightly concentrated around zero in these histograms, with mean values of 0.002 and 0.032 respectively. The mean values of the half-width at high contrast measured in physiology (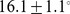) [47] and the model (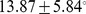) are also similar.

**Figure 8:**
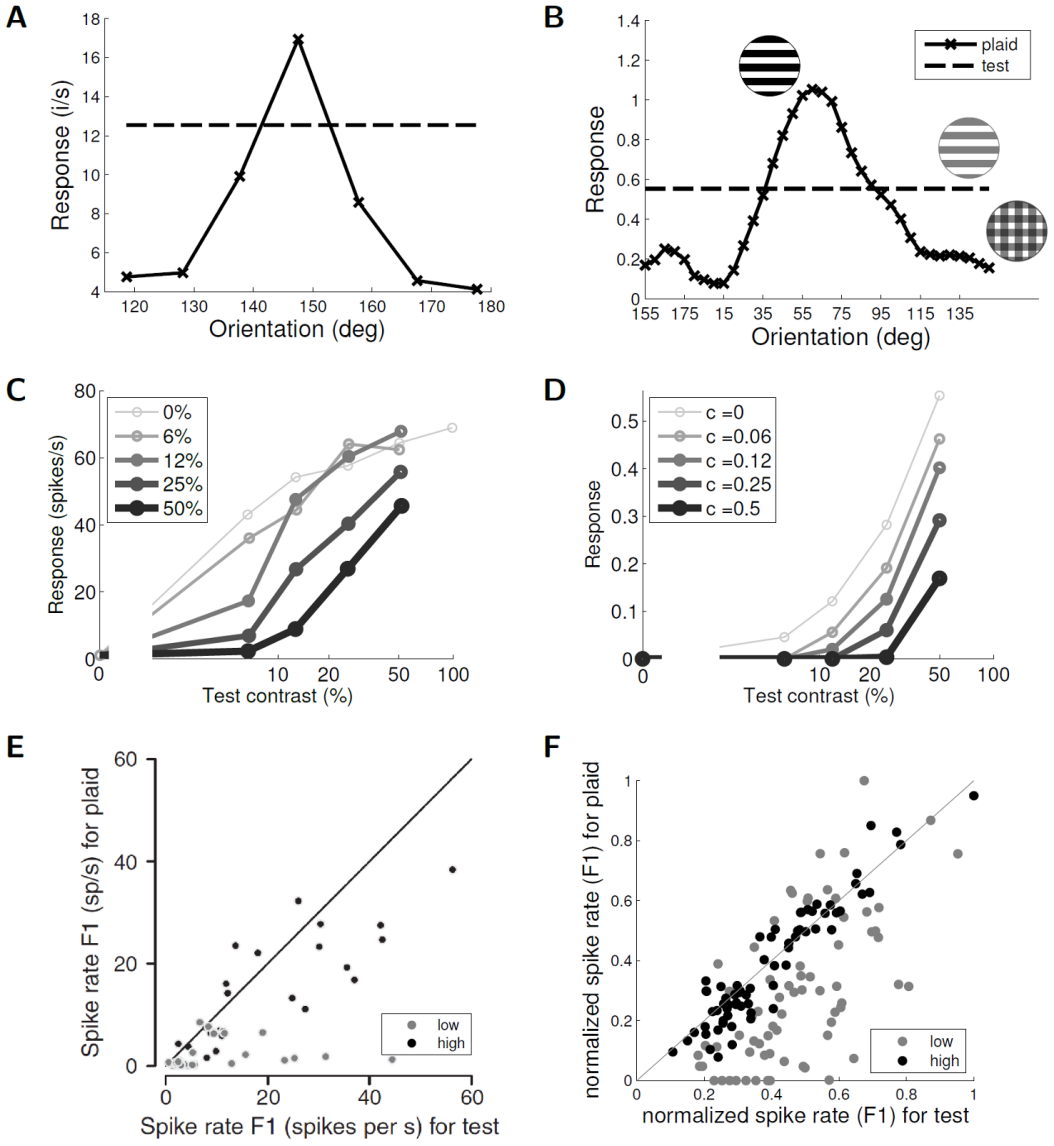
Cross orientation suppression. (A) A cat V1 simple cell demonstrates cross orientation suppression by responding with lower firing levels to an iso-oriented test grating if an ortho-oriented grating (mask) is superimposed (data replotted from [48], Figure 3(A)). The dashed line is the response to the iso-oriented test grating with no mask stimulus. (B) Cross orientation suppression exhibited by a sparse coding model neuron. Note the same dependence on the orientation of the mask that is seen in (A). (C) Contrast tuning curves of the test at different fixed mask contrast levels for a cat simple cell (data replotted from [50], Figure 2A). (D) Contrast tuning curves of the test for the same sparse coding model cell as in (B). Note again the same response modulation as in physiology despite the lack of contrast saturation in the model (see Discussions). (E) Measurement of modulation (F1) component of the response to a test grating alone vs. that with a superimposed orthogonal grating from a population of visual cortical neurons in cat (reprinted by permission from Macmillan Publishers Ltd: Nature Neuroscience, Figure 2b from [51]). The unity line represents where there is no suppression. The response at low test contrast is further away from the diagonal, suggesting more suppression in this regime. (F) Measurement of F1 response to a test grating alone vs. that with a superimposed orthogonal grating from the sparse coding model population. Note that just as in the physiology data, the model has the same general suppressive behavior, with increased suppression with lower test contrast.

An example of a nonlinear CRF effect using a more complex stimulus is *cross orientation suppression*, where a plaid (i.e., an ortho-oriented mask grating superimposed on an iso-oriented test grating) suppresses the response of the cell to the test alone. Fig. 8A and Fig. 8B show examples of this suppressive tuning property from cat V1 [48], as well as from a single cell in the sparse coding model. This kind of facilitatory effect may be due to a number of factors, including excitatory connections between cells (i.e., other cells in the population encouraging the target cell to represent the stimulus when they are unable to do so) or dis-inhibition, where a distant cell inhibits an intermediate cell that subsequently releases an inhibitory effect on the target cell [49].

The degree of cross orientation suppression depends on other factors beyond the orientation of the mask stimulus, including the contrast levels of the test stimulus. This contrast dependency was observed in cat V1 (shown in Fig. 8C) [50], and is also visible in the sparse coding model neurons as shown in Fig. 8D. Note that while the qualitative trends in the contrast dependency are the same in the model and in physiology, the lack of contrast saturation in the present model is evident in this figure (see Discussions).

The degree of cross orientation suppression expressed in a population of cells can be characterized by comparing the response to the plaid with the response to the test alone. A scatter plot of the normalized spike rate of cat V1 cells shown in Fig. 8E for the test versus plaid stimuli demonstrates that most cells have a suppressive response to the plaid (as depicted in the single cell response in Fig. 8A) [51]. Furthermore, the scatter plot indicates that the suppression is more pronounced for lower test contrasts. As shown in Fig. 8F, the sparse coding model population exhibits the same qualitative properties, with most cells exhibiting plaid suppression that increases with lower test contrast. Quantitatively, the mean cross orientation suppression ratio between the test and plaid responses for cat V1 was measured at 0.11 for low test contrast and 0.71 for high test contrast [51]. The sparse coding model cells have mean cross orientation suppression ratios of 0.59 and 0.95 for low and high test contrasts (respectively). While the model shows the same qualitative trend and overlaps in range, the specific values for these ratios are slightly higher than the reported experimental values. This small quantitative discrepancy might be due to the presence of contrast saturation in the physiology (visible in Fig. 8C) and its absence in the sparse coding model (Fig. 8D; (see Discussions).

## Discussion

Electrophysiology research in V1 has revealed a wide variety of nCRF effects that may appear to be due to many different aspects of neural coding or cortical processing. The functional interpretation of these effects is especially complex given the heterogeneity of the responses exhibited across populations of cells reported in the literature. We have demonstrated for a wide variety of nCRF effects that both the canonical individual cell response properties and a substantial diversity of population response properties are emergent characteristics of a simple dynamical system implementing a sparse coding model. This model appears to produce a very good qualitative match to many measures of population response statistics, and in many cases produces quantitative measures of these statistics that are in a similar range to reports in the physiology literature. By demonstrating a coding model that can account for these response properties, these results provide a potential functional insight into the role of nCRF effects in optimal sensory coding. While not mutually exclusive of other functional models that may also play a role in neural coding, the sparse coding model is one of the few models (along with [52]) able to substantially reproduce some nCRF effects as well as account for the emergence of localized, oriented, and frequency-selective CRFs [14]. In particular, despite not being constructed to produce nCRF effects, the present model appears able to capture population properties of nCRF effects that have been difficult for other functional models to produce (e.g. the contrast invariance of surround suppression index in Fig. 3F, as discussed in [53]).

There are several existing results that share a similar goal of providing high-level functional interpretation of nCRF effects. Perhaps most closely related to the present study is the PC/BC model [23], [52], [54]–[56], which has also been able to reproduce most of the nCRF effects demonstrated in this paper [23]. It is interesting to note that although it has other functional goals, the PC/BC model does exhibit high sparsity [52] and has accounted for classic CRF tuning properties [52]. While there is significant overlap in the demonstrated nCRF effects, the present work is unique in exhibiting the sufficiency of a model derived from sparse coding to produce the observed effects and to reproduce the population diversity seen in physiology (which the PC/BC model has yet to demonstrate). Given the similar behavior of the PC/BC model and the present model, it is possible that there is a deeper underlying relationship between the PC/BC model and sparse coding than is presently understood. Other example related works include the basic predictive coding model [57], where a subpopulation of model neurons communicating prediction errors exhibits some of the single cell nCRF effects documented in the present study. Another example is the divisive normalization model [58], where contextual effects emerge from a population interaction that modulates the gain in an attempt to maximize the independence of neighboring units. While both of these models account for some individual effects, they are not currently known to reproduce the population diversity seen in physiology or to alone be sufficient to also account for the emergence of known CRF properties (without an added sparsity constraint). More recent models capture the center-surround homogeneity (e.g. orientation co-alignment) in the natural scenes through a generalized form of divisive normalization [59] or capture the covariance structure between pixels in natural scenes [60]. While each of these models demonstrates some individual nCRF effects, these models are also not currently known to reproduce the population diversity seen in physiology (in particular, [59] simulates responses using a single generic unit and not a diverse population) and neither model currently has a fully specified implementation in a biologically plausible circuit (although an approximate form of the model in [60] may enable such an implementation). Another related model was described in [61], which demonstrated that a spiking input targeted divisive inhibition mechanism gives rise to competition among sensory feature detectors and non-classical-like effects. While this model have some interesting features that the present model does not have (e.g., biologically realistic spiking behavior), the stimuli and CRF representations were 1D idealized functions and it's not clear how the results extend to 2D images.

An important feature of the present work is that the same model (with the same parameters) is used to produce all of the presented results (i.e., parameters were tuned once and fixed for all experiments in the main text). The qualitative and quantitative matches observed in this paper rely on these parameter settings combined with the dynamical system implementation of the sparse coding rule. For example, changes in the system that would actually encourage responses with higher sparsity (e.g., increasing 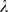, solving Eq. (2) using a conventional digital algorithm, running the dynamical system implementation with more integration time steps/faster non-biological time constants) would often generate similar single cell nCRF effects [62] as presented here (results not shown), but those effects would be too strong to be a quantitative match to the population properties (e.g., a far higher percentage of model cells would show strong surround suppression than is reported in physiology; see Fig. S1 which is described in Supporting Information Text S1). The Supporting Information demonstrates some instances where simple parameter changes in the model can actually account for apparently conflicting reports regarding nCRF effects in the experimental literature. We speculate that different settings of 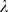 in the model may reflect differences in experimental preparations, such as different species and various levels of anesthesia. Indeed, anesthesia is known to influence the sparsity level in sensory systems [63], [64], and some perceptual contextual effects only occur in awake animals [65]. These observations about changes in the results with varying sparsity levels indicates that the sparse coding objective appears to be sufficient to produce the nCRF modulations, but the dynamical system implementation (with biophysical time constants) is required to produce the heterogeneity necessary to be a good quantitative fit. We also note that the role the dynamical system plays in the present work is similar to recent work [15] showing that learned dictionaries can be a much better quantitative match with measured macaque CRFs when the sparse coding model is implemented in a neurally-plausible network model. It is presently unclear if a different dynamical system minimizing the sparse coding objective would also result in the heterogeneity necessary to still be a good quantitative fit to physiology. Similar variations in the quantitative fits (especially to population data) are expected when using other sparsity penalties beyond the 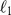 norm used here [28], or when using sparse coding implementations that encourage more “hard” sparsity (i.e., more elements that are exactly zero) [15]. In a similar vein, the present study uses a four-times overcomplete dictionary optimized for sparsity under natural scenes, and this model component is also likely important to the presented results. Though investigating the role of the dictionary would be an interesting avenue of further exploration, we expect that larger dictionaries may enable more sparse responses which also may demonstrate more suppression than what is seen in the current model.

**Figure 9:**
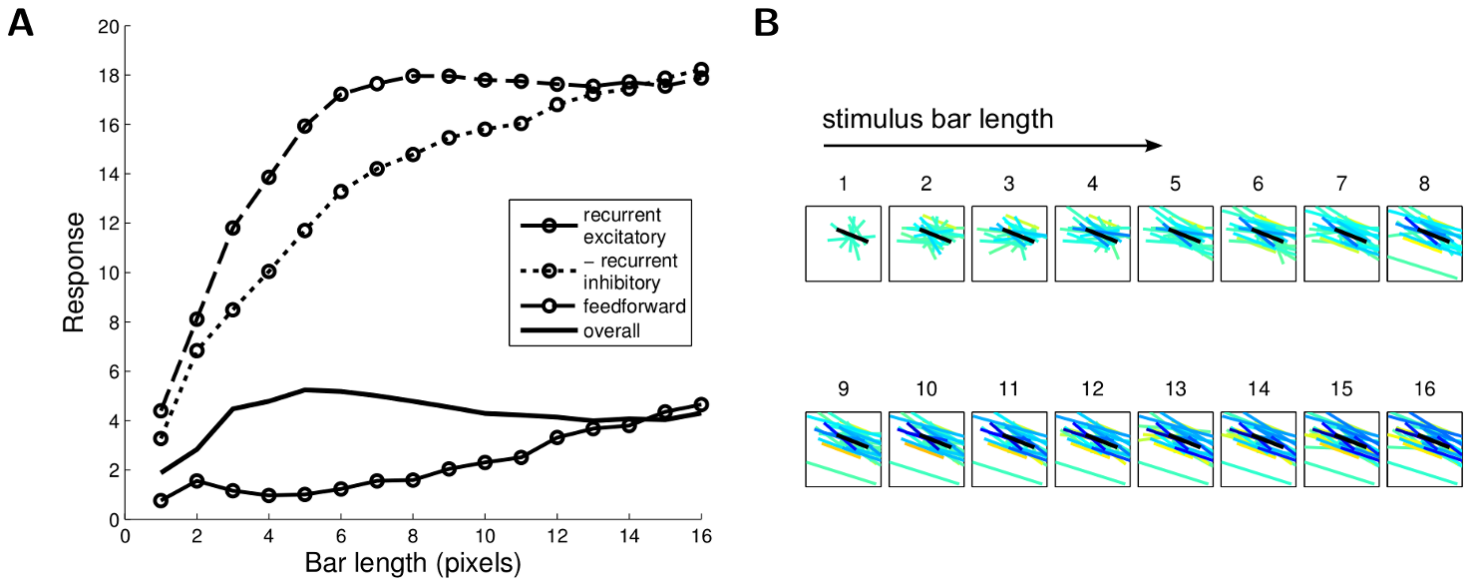
Decomposition of the recurrent inputs contributing to the end-stopping effect. (A) Overall decomposition of the response into recurrent excitatory, inhibitory, and feedforward components; (B) Locations and orientations of the CRFs of cells contributing to the recurrent excitatory and inhibitory signals at different bar lengths. Only CRFs with significant influences are displayed (i.e., 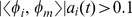 at steady state). The warmer color (yellow) represents the location and orientation of the CRFs for cells contributing to recurrent excitation, the cooler color (blue and cyan) represents the CRFs for cells contributing to recurrent inhibition. Higher contrast in the color indicates a stronger excitatory or inhibitory effect on the target cell. The black bar represents the target cell CRF. Note that as the bar length increases, the suppressive effect is mostly due to recurrent inhibition from cells that are a better description of the new stimulus (and therefore would be a more efficient stimulus description according to the sparse coding model).

The recurrent interactions between cells in the sparse coding model implement a rich nonlinear response where cells compete to represent stimulus features. While it has been noted that stimuli in the CRF surround can produce sparse responses [5], [6], [8], the surprising finding of this work is that the particular form of inhibition and excitation necessary to implement a sparse coding model is sufficient to explain so many individual and population nCRF properties. At a high level, these effects likely arise from the present model because the observed responses produce a more efficient representation of the stimulus than alternative population responses. While a detailed investigation of how the network interactions give rise to the response properties is an interesting open question for future investigation, in general this is difficult to determine due to the interactions between the network dynamics and the stimulus dynamics (i.e., the response properties arise from the average response over a drifting grating, in addition to being influenced by network dynamics). In the case of end-stopping, the stimuli is not drifting and we can see more explicitly how this effect arises from the principles of sparse coding. In response to a given fixed stimuli, the steady-state network response is composed of a combination of feedforward excitation, recurrent excitation and recurrent inhibition. When plotting these three components of the steady-state response as a function of the bar length (Fig. 9A), it is evident that the overall response is mostly driven by the feedforward component and the recurrent inhibition. The feedforward excitation saturates as a result of the stimulus growing out of the CRF, but the recurrent inhibition keeps growing with increased bar length. To see the spatial extent of the recurrent influence, Fig. 9B shows the CRF locations and orientations of the cells influencing the target cell. As expected, inhibition mostly comes from cells with overlapping and co-linear CRFs that represent a more efficient description of the stimulus as the bar length increases.

There has been a long history of debate over the mechanisms underlying various nCRF effects [3], with each effect generally having a substantial literature attempting to answer questions about the detailed aspects underlying the modulatory response properties (e.g., the relative role of intra-cortical connections versus feedforward projections from thalamus in contrast invariant orientation tuning [66], as well as the role of feedback connections [67]). The implementation used in this work (see Materials and Methods) would appear to suggest that these contextual effects can emerge from recurrent network structure in the absence of nonlinearities in the thalamic input or feedback from higher cortical areas. However, mechanistic interpretation of functional models must be cautious as there are often many possible mappings of the model to circuitry and biophysical mechanisms. For example, past work has shown that it is possible to have mappings of functional models onto circuitry that are very different from their original intuitive mappings (e.g., divisive normalization [68] and predictive coding [54]). The sparse coding dynamical system used in this study is open to the same variety of mechanistic interpretations. For example, the recurrent inhibitory influences could be implemented [69] via local inhibitory interneurons receiving convergent inputs from local excitatory neurons [70] and having dense (many-to-one) output connections with these excitatory neurons [71]. Alternately, it is possible that these inhibitory influences could be implemented via a mechanism based on long term depression of synaptic connections between excitatory cells in cortical layer 4 [72] and global inhibition [73]. For another example, as demonstrated in [68], it might be possible to achieve similar computational goals through nonlinearities in the feed-forward thalamocortical circuit, rather than a recurrent network. For yet another example, the recurrent competition could be implemented through subtraction as in our model, or through division as in [23]. It remains an open question to determine the most biophysically appropriate mapping of the present model onto a circuit implementation.

**Figure 10:**
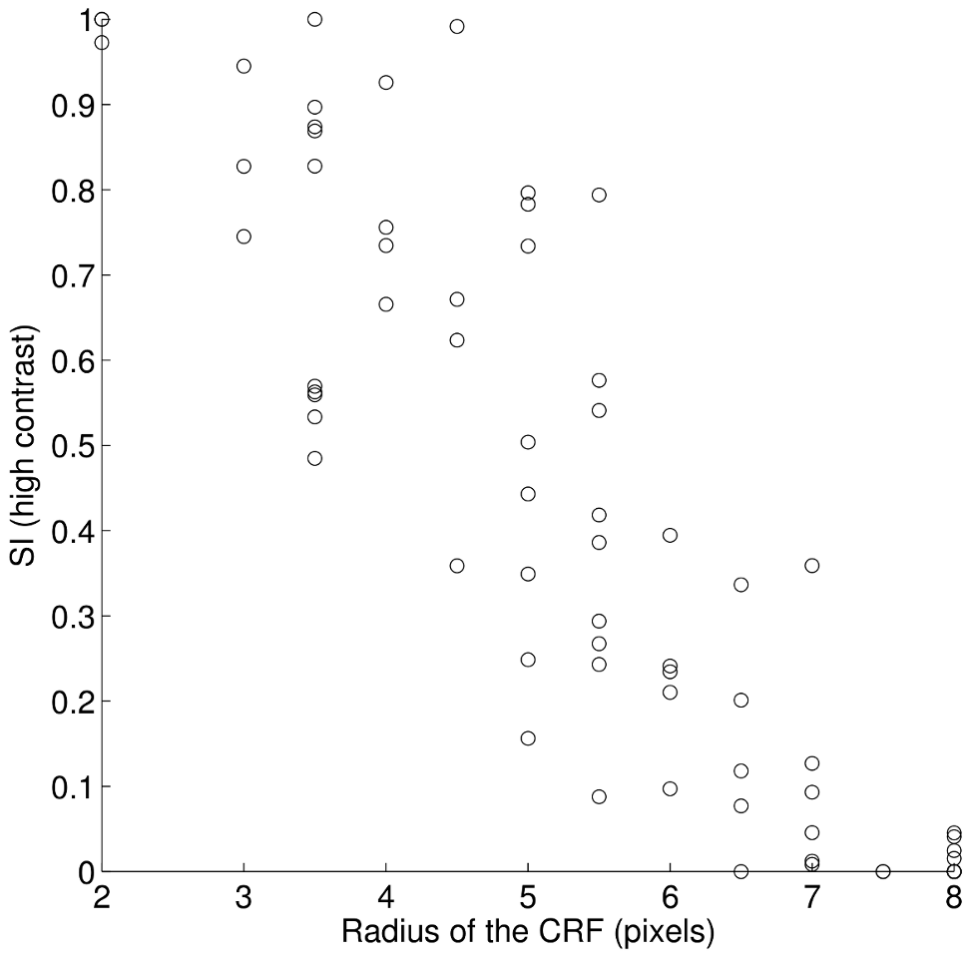
Surround suppression index is anti-correlated with the CRF size. Cells with larger CRFs tend to be less suppressed by a surround stimulus (correlation coefficient 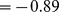; 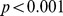). The level of suppression is measured by the suppression index (SI) at high stimulus contrast.

While the mechanisms underlying individual nCRF effects is an interesting area of investigation, another related question of interest is to determine which aspects of the model are responsible for the observed population variability. In the present model, the dictionary serves to define both the activity driving each cell through the CRF, as well as determining the synaptic weights that define the recurrent influences in the network dynamics. Because the present dictionary was learned from the sparse coding objective on natural images, it is optimal for this coding strategy and demonstrates significant variability as observed in biological CRFs. While a detailed investigation of how the model gives rise to the response diversity is also a challenging and interesting open question for future investigation, one interesting preliminary question is what role the variability in the dictionary plays in the observed nCRF response variability. As a specific example, we have found the surround suppression index to be significantly anti-correlated with the CRF size (Fig. 10; correlation coefficient 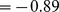; 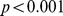). While we are unaware of studies investigating this relation in the physiology literature, there are several studies that do suggest this type of anti-correlation. One piece of evidence [74] shows that cortical layers with larger CRFs also tend to have lower SIs and vice versa. Another corroborating study [75] shows that suppressive V1 cells have smaller CRFs compared to plateaued and facilitative cells. This anti-correlation may be present simply because there are fewer cells with larger CRF size in the model (visible in Fig. 10) and in V1 [76], making these cells more likely to be used in an efficient coding model whenever the stimulus grows past a certain size. It is also possible that the limited stimulus sizes used in the current model and many physiology studies (e.g. [77]) could be producing a boundary effect that contributes to some of these observations. It is presently unclear if the inherent variability in the dictionary is alone sufficient to produce the response variability observed in biology (i.e., if another coding model could produce this same variability when using CRFs from this same type of learned dictionary) or if significant response heterogeneity requires the interaction of a learned dictionary with a dynamical system implementing sparse coding.

Some contextual effects, especially ones that involve perception such as perceptual pop-out, figure ground segregation [13], and contour integration [12] operate over a larger range (e.g. over 8 times the CRF size in [78]) and are likely to be mediated by long-range lateral connections [79]. The present study did not test the emergence of these types of effects in the sparse coding model due to the limited size of the dictionary elements. The sparse coding model simulated here used a substantially overcomplete dictionary (see Materials and Methods), thus the size of the visual field we were able to simulate is limited by the current computational complexity of learning large scale dictionaries from the statistics of natural images. While it may seem unlikely that long-range effects could emerge from the present model when the only direct influences are between cells with overlapping receptive fields (see Materials and Methods), it is conceivable that second order effects (e.g., dis-inhibition, where a distant cell inhibits an intermediate cell that subsequently releases an inhibitory effect on the target cell) may play a central role that would only be discovered in a study using larger visual fields. An alternative is to incorporate long-range lateral connections explicitly into a sparse coding model [80].

Despite the wide variety of nonlinear properties observed in the sparse coding model, this model alone is unable to reproduce some nCRF effects because it lacks the stereotypical saturating contrast response function [81]. While this contrast saturation would be a simple addition to the model, the present study focuses on the basic sparse coding model to isolate the response properties due to the nonlinear interactions required to achieve sparsity. It is interesting to note that the model can still reproduce several contrast dependent contextual effects even without an explicit contrast saturation mechanism. Indeed, it has been previously suggested that some of these contrast dependent effects may be independent of the response saturation [42]. Nevertheless, we expect that including some type of contrast saturation in the model may improve the quantitative fit of the current model to some nCRF effects. For example, introducing contrast saturation in the surround suppression simulation (Fig. 3) may further restrict the size tuning curve peak at high contrast and lead to a closer match to the expansion ratios reported in the physiology literature. Contrast saturation could be included in this model through several mechanisms, including modifying the cost function to encourage saturating spike rates (although by itself this mechanism may not accurately capture saturating membrane potentials [82]), including LGN saturation [54], modifying the network implementation to include contrast-dependent shunting inhibition [4], or coupling the sparse coding model with a model such as the previously reported divisive normalization [58].

## Materials and Methods

To implement sparse coding in a neurally plausible network architecture, we solve the dynamical system in equation (3) using a first order Euler method with an integration time step of 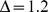
ms, 25 integration time steps per stimulus (i.e., corresponding to a stimulus presentation of approximately 1/30 second per frame of a video), a sparsity level of 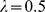 and a membrane time constant of 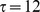
ms (within the range of physiological values between 10 ms and 100 ms [83]). In simulations using static stimuli we measured the response after 1000 integration time steps to assure full convergence.

Stimuli such as bars and sinusoid gratings were generated as 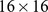 pixel image patches, whitened (to mimic retinal processing), and overlaid on a gray background with the same mean as the gratings. Finally, for all stimuli we used a contrast (defined as the range of the intensity values of the sinusoid grating or bar) of 0.3 unless otherwise noted.

As in physiological experiments studying nCRF effects (e.g. [36]), we first picked an arbitrary “target” neuron from the population that we would “record” from, pinpointed the center of its CRF ON-region by hand (interpreting the dictionary element as approximating the CRF), and searched for an optimal circular sinusoidal static grating patch stimulus (i.e., having the size, orientation, spatial frequency, and phase that gave rise to the maximal response of the target neuron in the model). We performed this search by a two-step exhaustive search over the parameter space using the following ranges: size of the grating was between 1 pixel and 16 pixels in diameter using 0.5 pixel increments; orientation was between 0 and 175 degrees using 5 degree increments; spatial frequency was between 0.5 to 2 radians/pixel using 0.25 radians/pixel increments; phase was between 0 to 2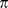 using 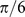 radian increments. We used this approach to map the optimal stimuli for a total of 72 simulated cells (each with CRFs well-localized within the limited visual field used in the simulation).

In most experiments we used drifting sinusoid gratings as stimuli (as described in the experimental literature for each effect). We simulated a drifting grating in discrete time by a series of static gratings at progressive phases. We fixed the temporal frequency of the grating to be about 3 Hz, which is typical of the preferred frequency of cortical neurons [83]. To simulate the dynamic effect of the neural response, we simulated the dynamical system in equation (3) through the entire experiment with the driving input switched at the appropriate time to match the drift speed of the grating. We measured the response to a full cycle of the grating presentation by the mean or F1 (first harmonic) component, depending on the measure used in physiology literature for the particular effect under consideration.

In the end-stopping experiment we found an optimal static bar stimulus for the target neuron by fixing the bar width to 2 pixels, the orientation to be the same as the optimal sinusoid grating orientation, and the bar length to be the same as the optimal grating size. We then found the optimal bar location by translating the bar around a 5-pixel neighborhood of the grating center and searching for the maximal model response for that cell. After the optimal bar stimulus location was found, we increased its length from 1 to 16 pixels and recorded the steady-state response from the model.

In the surround suppression simulation, we varied the contrast of the sinusoid grating stimuli from 0.05 to 0.5 with increments of 0.1, and we varied the size from 1 to 16 pixels in diameter with an increment of 1 pixel (other parameters were fixed). We measured the spike rate in response to the drifting grating by the F1 component. We defined the surround suppression index as 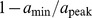, where 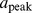 represents the peak response across all stimulus sizes at a certain contrast, and 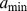 represents the minimum response at a radius larger than the peak. Response to high contrast was measured at 0.5 and low contrast at 0.05.

In all orientation tuning studies, we stepped the orientation of the stimulus from 0 to 180 degrees in increments of 5 degrees. We measured the mean spiking response to the drifting grating. When studying the contrast invariance property, we stepped the contrast from 0.1 to 0.5 in increments of 0.1. In the population study of the tuning width, we measured tuning curve half-width at half-height by 1.17 times the standard deviation of the Gaussian fit to the orientation tuning curves. When measuring the slope of half-width vs. contrast, we normalized the contrast to 100 [47]. Five neurons in the simulated population had small unipolar CRFs and therefore showed very little orientation tunings. We could not fit Gaussians successfully to the tuning curves for these neurons, and therefore did not include their orientation tuning properties in the population study.

In the center surround orientation tuning experiment, the surround annulus grating had a thickness of 2 pixels and the center and the surround were phase-locked. When measuring the surround orientation tuning, we fixed the center orientation at the optimal orientation and measured the response to the center alone as well as the center plus the surround. We measured the response measurement for two different center radii: the optimal and the optimal plus one pixel. In the experiment that studied the contrast's effect on the center surround orientation tuning, the center contrast took on values on a logarithmic scale (0, 0.03, 0.06, 0.12, 0.25, 0.5) and we kept the surround contrast constant at 0.5. Similar to the observation in physiology (Fig. 8E), there are many cells with weak response at low contrast in the simulation. Due to the present simulation having more cells than the study in [48], this clustering around zero made the low contrast responses difficult to read when plotted. To better visualize the suppression effect of the plaid for weakly responsive neurons, we plotted the low-contrast population responses with the maximum response normalized to 1 (effectively spreading the points out over the full range to better see their position above or below the diagonal line). High-contrast responses were similarly normalized to plot on the same scale.

## Supporting Information

Figure S1 **Surround suppression index distribution under a different parameter setting.** Related to Fig. 3 in the main text and discussed in Supporting Information Text S1. With steady-state response of the model and otherwise default parameters, the surround suppression index distribution shows physiologically unrealistic large percentage of cells with complete suppression.
(TIF)

Figure S2
**Surround suppression index distribution under another parameter setting.** Related to Fig. 3 in the main text and discussed in Supporting Information Text S1. (A) Physiologically measured index from an experiment on macaque monkeys (N = 105); data replotted from [38], Figure 2C; (B) Simulation of the surround suppression index distribution with lower sparsity and longer convergence times (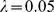 and 1000 integration time steps). Note that the majority of neurons are surround suppressive in this case.(TIF)

Figure S3
**Facilitatory influence.** Related to Fig. 6 in the main text and discussed in Supporting Information Text S1. (A) Facilitatory influence from the iso-surround at low center contrast observed in cats; data replotted from [44], Figure 5; (B) A simulated neuron demonstrates a similar effect when the tradeoff parameter is set to 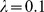.(TIF)

Figure S4 **Spatial organization of surround orientation tuning.** Discussed in Supporting Information Text S1. Orientation tuning with “gap” in between center and surround. (A) Physiology without gap; data replotted from [41], Figure 4D; (B) Simulation without gap; (C) Physiology with gap; data replotted from [41], Figure 4E; (D) Simulation with gap. Parameters same as in Fig. 5 in the main text.
(TIF)

Text S1 **Effects of changing simulation parameters and miscellaneous nCRF effects.**
(PDF)
