# Supplementary figures and images for: Visual Nonclassical Receptive Field Effects Emerge from Sparse Coding in a Dynamical System
Source: PLoS Comput Biol. 2013 Aug 29;9(8):e1003191. doi: 10.1371/journal.pcbi.1003191 (PMC3757072; doi:10.1371/journal.pcbi.1003191)

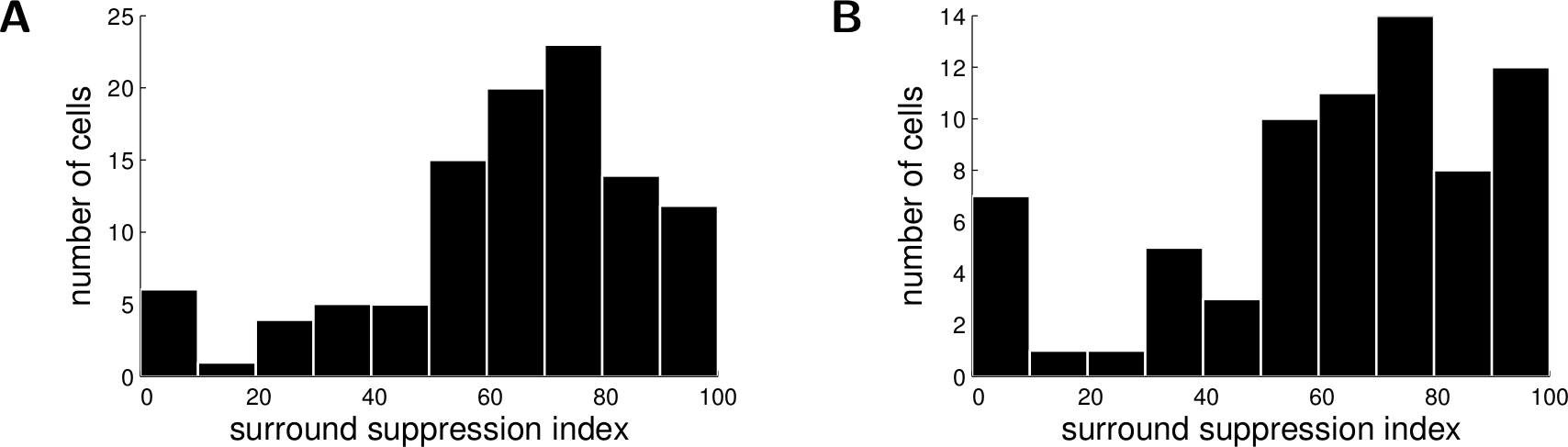

Supplement: Figure S2 — Surround suppression index distribution under another parameter setting. Related to Fig. 3 in the main text and discussed in Supporting Information Text S1. (A) Physiologically measured index from an experiment on macaque monkeys (N = 105); data replotted from [38], Figure 2C; (B) Simulation of the surround suppression index distribution with lower sparsity and longer convergence times ( and 1000 integration time steps). Note that the majority of neurons are surround suppressive in this case. (TIF) [file pcbi.1003191.s002.tif]

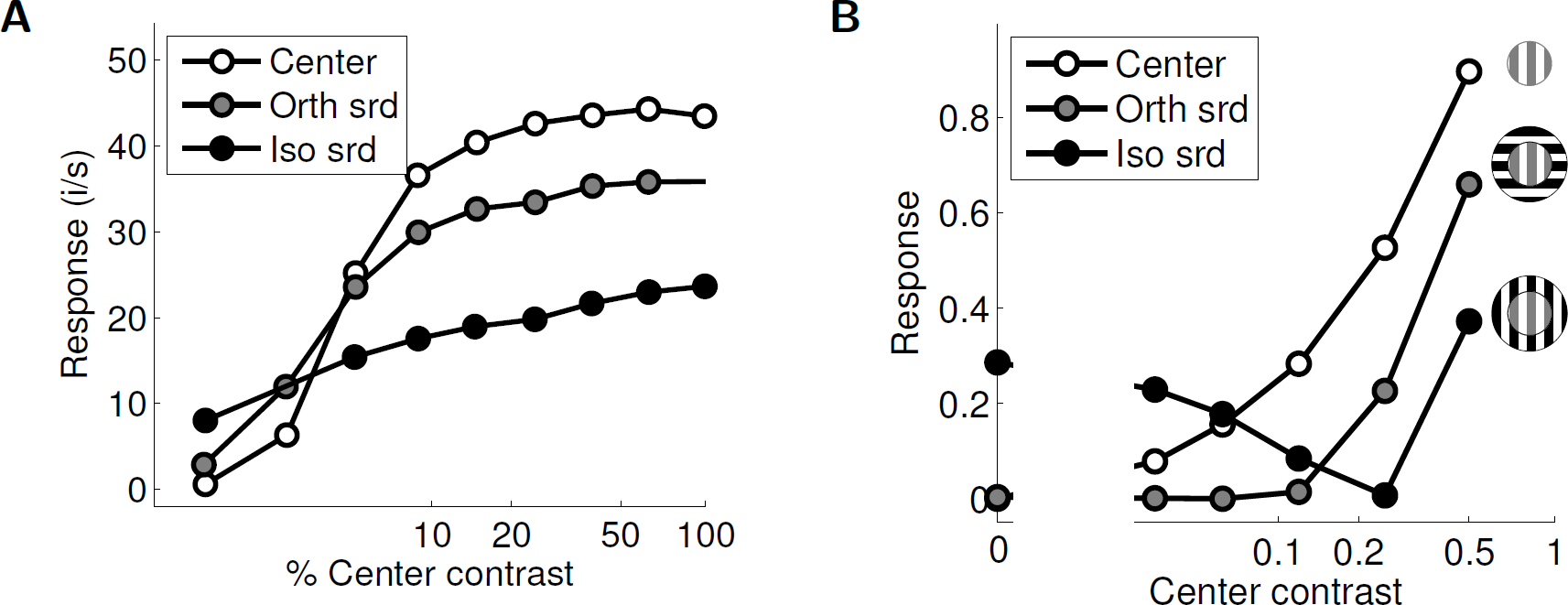

Supplement: Figure S3 — Facilitatory influence. Related to Fig. 6 in the main text and discussed in Supporting Information Text S1. (A) Facilitatory influence from the iso-surround at low center contrast observed in cats; data replotted from [44], Figure 5; (B) A simulated neuron demonstrates a similar effect when the tradeoff parameter is set to . (TIF) [file pcbi.1003191.s003.tif]

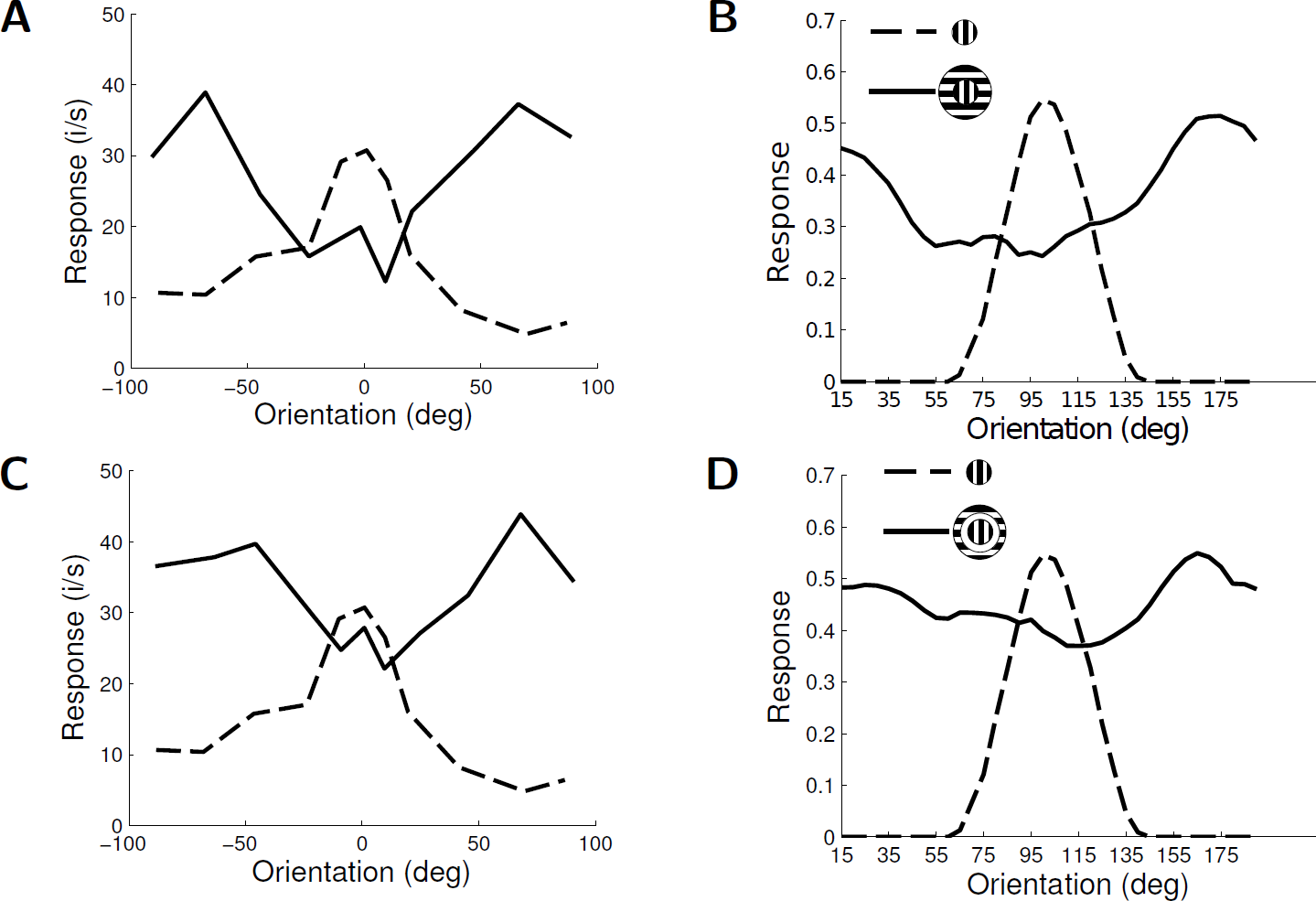

Supplement: Figure S4 — Spatial organization of surround orientation tuning. Discussed in Supporting Information Text S1. Orientation tuning with “gap” in between center and surround. (A) Physiology without gap; data replotted from [41], Figure 4D; (B) Simulation without gap; (C) Physiology with gap; data replotted from [41], Figure 4E; (D) Simulation with gap. Parameters same as in Fig. 5 in the main text. (TIF) [file pcbi.1003191.s004.tif]
