# Supplementary material for: Visual Nonclassical Receptive Field Effects Emerge from Sparse Coding in a Dynamical System
Source: PLoS Comput Biol. 2013 Aug 29;9(8):e1003191. doi: 10.1371/journal.pcbi.1003191 (PMC3757072; doi:10.1371/journal.pcbi.1003191)
Supplement: Text S1 — Effects of changing simulation parameters and miscellaneous nCRF effects. (PDF) [file pcbi.1003191.s005.pdf]

Supporting Information: Text S1

Mengchen Zhu[1], Christopher J. Rozell[2,*]
1 Mengchen Zhu: Wallace H. Coulter Department of Biomedical Engineering, Georgia Institute of Technology, Atlanta, GA 30332 USA;
2 Christopher J. Rozell: School of Electrical and Computer Engineering, Georgia Institute of Technology, Atlanta, GA 30332 USA.
∗ E-mail: crozell@gatech.edu

Effects of Changing Simulation Parameters

As mentioned in the main text, changing parameters such as the sparsity level λ and the simulation time changes the quantitative results for the simulation. For example, when simulating the dynamical system all the way to a steady state response (1000 integration time steps), the surround suppression index distribution shows an unrealistically large number of cells that demonstrate essentially complete suppression (Fig. S1; compare to Fig. 3D in the main text).

While such parameter changes can sometimes show biophysically unrealistic responses (especially for population statistics), these parameter changes can sometimes account for apparently conflicting reports in the physiology literature. For example, Fig. S2 shows the surround suppression index produced by the model when we lowered the tradeoff parameter λ (i.e., allowing more simultaneously active cells) and increased the number of integration time steps for the system (i.e., presenting the stimulus to the system for a longer time, thereby letting the network converge more fully). In this case, the model produced a surround suppression index that had most cells being suppressive, peaking near a value of 70%. Interestingly, this is qualitatively similar to other reports from the physiology literature [1–3] that are apparently conflicting with the more typical reports of most cells being non-suppressive, perhaps due to a different experimental preparation.

For another example of a case when a different set of parameters could be used to replicate an alternative report from the literature, consider the fact that some neurons in cat and macaque V1 actually show facilitation with iso-oriented surround stimuli when using low center contrast stimuli [4, 5] (Fig. S3**A**; not observable in Fig. 6D in the main text). Such effects were previously modeled mechanistically [5–7] as a result of changing balance between the excitation and inhibition under different contrast (input strengths). We show that this facilitation effect also emerges from our functional model response when the tradeoff parameter is set to $\lambda = 0.1$ and all other stimulation procedures are kept the same (see Fig. S3). This might be understood as a result of the nonlinear change of competition between neurons with different contrast/input drives (potentially with secondary effects such as disinhibition). Related to our result, Coen-Cagli et al. [8] recently showed that surround facilitation could arise under certain stimulus conditions in a statistical model adapted to natural scenes. In their model, how much the center response is normalized (modulated) by the surround response is dependent on the relative stimulus contrast in the center and surround, as well as the receptive field correlations between the center and surround (similar to the present model, see Discussion section in the main text for more details on the differences). We note that contrast-dependent facilitation might be beneficial for perceptual tasks such as contour integration. For example, when the

center contrast is low, it is perceptually helpful to enhance the response to fill in the gap and complete the contour. However, when the center contrast is high, it is more efficient to keep the response in check and relegate some of the responsibility for the representation to other cells.

Other Miscellaneous nCRF Effects

Using a similar setup as in Fig. 5 in the main text, the suppressive effect of the surround is smaller if there is a gap separating it from the center (Fig. S4). The extent of contextual effect is therefore modulated by the area the surround is covering. The larger the surround, the stronger the effect.

References

1. Jones H, Grieve K, Wang W, Sillito A (2001) Surround suppression in primate V1. Journal of Neurophysiology 86: 2011–2028.

2. Sceniak MP, Hawken MJ, Shapley R (2001) Visual spatial characterization of macaque v1 neurons. Journal of Neurophysiology 85: 1873-1887.

3. Adesnik H, Bruns W, Taniguchi H, Huang ZJ, Scanziani M (2012) A neural circuit for spatial summation in visual cortex. Nature 490: 226–231.

4. Somers D, Todorov E, Siapas A, Toth L, Kim D, et al. (1998) A local circuit approach to understanding integration of long-range inputs in primary visual cortex. Cerebral Cortex 8: 204–217.

5. Ichida J, Schwabe L, Bressloff P, Angelucci A (2007) Response facilitation from the" suppressive" receptive field surround of macaque V1 neurons. Journal of Neurophysiology 98: 2168–2181.

6. Schwabe L, Obermayer K, Angelucci A, Bressloff P (2006) The role of feedback in shaping the extra-classical receptive field of cortical neurons: a recurrent network model. Journal of Neuroscience 26: 9117.

7. Ahmadian Y, Rubin DB, Miller KD (2012) Analysis of the stabilized supralinear network. ArXiv e-prints .

8. Coen-Cagli R, Dayan P, Schwartz O (2012) Cortical surround interactions and perceptual salience via natural scene statistics. PLoS Comput Biol 8: e1002405.

9. Jones H, Wang W, Sillito A (2002) Spatial organization and magnitude of orientation contrast interactions in primate V1. Journal of neurophysiology 88: 2796–2808.
